# Fungicidal effects of eight disinfectants against resistant *Candidozyma auris* (formerly *Candida auris*), *Aspergillus fumigatus*, and *Trichophyton indotineae*

**DOI:** 10.1128/spectrum.01055-26

**Published:** 2026-09-25

**Authors:** Hailin Zheng, Dongmei Li, Xiaofang Li, Naicen Ge, Huan Mei, Yuping Chen, Guanzhao Liang, Xiaodong She, Weida Liu

**Affiliations:** 1 Hospital for Skin Diseases, Institute of Dermatology, Chinese Academy of Medical Sciences and Peking Union Medical College 12501, Nanjing, China; 2 Jiangsu Provincial Key Laboratory of Dermatology, Nanjing, China; 3 Department of Microbiology and Immunology, Georgetown University Medical Center 12231, Washington, DC, USA; USDA-ARS-NPRL, Dawson, Georgia, USA

**Keywords:** disinfectants, nosocomial infections, resistant fungi, minimum fungicidal concentration, fungicidal activity

## Abstract

Disinfectants are widely used in hospital environments to control bacterial, viral, and fungal hospital-acquired infections. In recent years, nosocomial infections caused by drug-resistant fungi, such as *Candidozyma auris*, *Aspergillus fumigatus*, and *Trichophyton indotineae*, have posed an increasing transmission risk among patients and healthcare workers, making treatment more challenging. Although many disinfectants are routinely applied in hospital settings, data on their efficacy against drug-resistant fungi remain limited. In this study, we evaluated the antifungal efficacy of eight disinfectants against these three species of drug-resistant fungi, including aldehyde-, alcohol-, chlorine-, iodine-, and peroxide-based formulations. Using minimum fungicidal concentrations, all disinfectants exhibited fungicidal activity against the three fungi at appropriate concentrations; however, when tested at the manufacturer-recommended working concentrations using a quantitative suspension test in the presence of 0.3% bovine serum albumin as an organic interferent, only five disinfectants produced a ≥5 log_10_ reduction in viable organisms within 5 min of exposure. Comparative analysis further revealed that a commercial benzalkonium chloride solution (Meifute solution) and peracetic acid exhibited particularly strong potential for the rapid elimination of these drug-resistant fungal pathogens.

**IMPORTANCE:**

Disinfectants are primarily developed against bacteria and viruses; there are no dedicated fungicides for use in hospitals, especially for drug-resistant fungi. This study aimed to identify the best disinfectant among those already labeled as having fungicidal attributes for eliminating drug-resistant fungi, to address disinfection and infection control in medical institutions.

## INTRODUCTION

Fungi are globally ubiquitous in the environment (1–3). Approximately 60,000 fungal types are capable of infecting humans and other animals (4). Frequent contact with environmental sources increases the risk of fungal infection, particularly in immunocompromised hosts (5). In enclosed healthcare environments, including hospital wards and outpatient clinics where patients and healthcare workers interact closely, healthcare-associated infections (HAIs) are more likely to occur (6–8). The risk of pathogen transmission is further increased when drug-resistant microorganisms emerge, as they are more difficult to eliminate and can persist in healthcare environments despite routine infection control measures.

Outbreaks of fungal infections have been increasingly reported in healthcare settings, including *Candidozyma auris* outbreaks in nursing homes and *Aspergillus* infections among hospitalized patients (9–11). These pathogens often persist in hospital environments for extended periods, ranging from several hours to several months, and their transmission is easily facilitated by environmental factors (12). The distribution and relative abundance of fungal species vary across different clinical areas, with *Aspergillus* species among the most frequently isolated fungi in hospital wards (13). Increasing evidence indicates a rising prevalence of antifungal resistance in *Aspergillus* species. Several factors have been implicated in the emergence and spread of antifungal resistance, including empirical antifungal therapy, widespread agricultural use of azole-based pesticides, and environmental changes such as global warming. Effective environmental disinfection and the elimination of pathogenic fungi in hospitals and other high-risk settings are critically important for preventing nosocomial infections.

In this study, we selected three clinically important acquired drug-resistant fungal pathogens that pose significant challenges to clinical management: azole-resistant *C. auris*, multidrug-resistant *Trichophyton indotineae*, and azole-resistant *Aspergillus fumigatus*. Sensitive and drug-resistant strains were included in comparing the efficacy of different disinfectants; naturally drug-resistant strains such as *Fusarium* spp. or *Scedosporium* spp. do not have corresponding sensitive strains, so they were not examined.

Disinfectants widely used in medical environments in China include chlorine-based disinfectants, phenols, alcohols, and peroxides. Chlorine-containing disinfectants are primarily composed of sodium hypochlorite; they are commonly used in clinical practice and exhibit strong bactericidal and fungicidal effects (14). For example, 5% sodium hypochlorite solution effectively kills biofilms formed by *Candida albicans* (15), while 2.5% can inactivate the spores and biofilms of *Fusarium* and *Aspergillus* species (16), and 1% is sufficient to eliminate all spores of *Microsporum canis* and *Trichophyton* species (17). Phenol-based disinfectants (5%) also demonstrate strong activity against *Candida* species (18). Additionally, 2-(benzylideneamino) phenol has been identified as a promising hydroxyaldimine with potent antifungal activity against dermatophytosis (19). Ethanol-based disinfectants show variable efficacy depending on concentration; 40% ethanol achieved a 4 log_10_ reduction in all tested *Candida* strains (20), while 80% (vol/vol) ethanol inactivated *Aspergillus niger* ATCC 6275 and *Aspergillus brasiliensis* ATCC 16404 within 1 min (21). Hydrogen peroxide exhibits exceptional antifungal activity, achieving the highest log and percentage reduction against *A. niger* compared with other disinfectants (22). Although a wide variety of disinfectants are commercially available, research on their effectiveness against drug-resistant fungal strains remains limited.

Whether in hospitals or other environments, the proliferation of fungi, particularly drug-resistant strains, poses a significant threat to human health. Attention has increasingly focused on drug-resistant *C. auris*, *T. indotineae*, and *A. fumigatus. C. auris* and *A. fumigatu*s are included in the World Health Organization’s fungal priority pathogens list, and invasive infections caused by drug-resistant fungi are associated with high mortality rates (23). Effective use of disinfectants to eliminate these pathogens is critically important. In this study, we aimed to evaluate the fungicidal efficacy of eight disinfectants commercially available in China against drug-resistant *C. auris*, *A. fumigatus*, and *T. indotineae* to identify the most effective agents for controlling environmental fungal contamination. The selected disinfectants represent a range of formulations, including aldehyde-, alcohol-, chlorine-, iodine-, and peroxide-based disinfectants. Efficacy tests were conducted according to the Chinese national standard “Test Method for Bactericidal Effect of Disinfectants in Laboratory” (GB/T 38,502-2020).

## MATERIALS AND METHODS

### Strains and growth

A total of 30 strains were tested, including 10 isolates each of *A. fumigatus*, *C. auris*, and *T. indotineae*. All strains were obtained from the Medical Fungi sub-center of the Medical Sciences Pathogenic Microorganism Species Preservation Center of the Chinese Academy. These strains were initially identified based on morphological characteristics and subsequently confirmed through sequencing of the conserved ribosomal internal transcribed spacer and large subunit regions. DNA fragments encoding SQLE of the 30 strains were amplified using previously reported primers (24).

### Antifungal agents

Fluconazole, itraconazole, voriconazole, posaconazole, and terbinafine were all purchased from Sigma-Aldrich Co. LLC (USA). The inhibitory concentration ranged from 0.5 to 256 μg/mL for fluconazole, 0.03–16 μg/mL for itraconazole, 0.015–8 μg/mL for voriconazole and posaconazole, and 0.03–16 μg/mL for terbinafine.

### Disinfectant solution preparation

Eight disinfectants commonly used in China were tested. Active components and concentrations are summarized in Table 1. Initial solutions were as follows: 84 Disinfectant: 34–46 g/L sodium hypochlorite (CAS: 7681-52-9); dilute glutaral solution: 5 g/L glutaraldehyde (CAS: 111-30-8), 5 g/L chlorobutyl bromide (CAS: 6940-78-9); 75% ethanol: 75% ethanol (CAS: 64-17-5); iodophor: 4.2–5.2 g/L chlorhexidine acetate (CAS: 56-95-1), 0.9–1.2 g/L iodine (CAS: 7553-56-2), and 60%–70% ethanol (CAS:64-17-5); benzalkonium bromide: 30 g/L benzalkonium bromide (CAS: 7281-04-1); Meifute solution: 2.25 g/L benzalkonium chloride (CAS: 63449-41-2), silver ions (CAS: 14701-21-4), alkyl glucoside (CAS: 110615-47-9), menthol (CAS: 2216-51-5), citric acid (CAS: 77-92-9), lactic acid, glycerin (CAS: 50-21-5), coconut fatty acid amine (CAS: 61788-46-3); chlorhexidine: 0.2% chlorhexidine gluconate (CAS: 18472-51-0); and peracetic acid solution: 15%–18% peracetic acid (CAS: 5699-44-5), 12%–14.5% acetic acid (CAS: 64-19-7), and 24.2%–28.2% hydrogen peroxide (CAS: 7722-84-1). For drug susceptibility testing, each stock solution was serially diluted in a twofold gradient down to working concentrations of 1:1, 1:2, 1:4, 1:8, 1:16, 1:32, 1:64, 1:128, 1:256, 1:512, 1:1,024, 1:2,048, 1:4,096, 1:8,192, and 1:16,384.

**TABLE 1:** The trade names, active ingredients, and concentrations of eight disinfectants

Disinfectant/trade name	Main active ingredients and concentrations	The working concentration specified in the manual for use	Country
84 Disinfectant solution	34–46 g/L sodium hypochlorite	The original solution was diluted 400 times	China
Dilute glutaral solution	5 g/L glutaraldehyde, 5 g/L chlorobutyl bromide	The original solution was diluted 1,000 times	China
75% alcohol	75% ethanol	The original solution has not been diluted	China
Iodophor	4.2–5.2 g/L of chlorhexidine acetate, 0.9–1.2 g/L of iodine, 60%–70% ethanol	The original solution has not been diluted	China
Benzalkonium bromide	30 g/L benzalkonium bromide	The original solution was diluted 15 times	China
Meifute	2.25 g/L benzalkonium chloride, silver ions, alkyl glucoside, menthol, citric acid, lactic acid, glycerin, coconut fatty acid amine	The original solution has not been diluted	China
Chlorhexidine	0.2% chlorhexidine gluconate	The original solution has not been diluted	China
Peracetic acid	15%–18% peracetic acid, 12%–14.5% acetic acid, 24.2%–28.2% hydrogen peroxide	The original solution was diluted 60 times	China

### Clinical and Laboratory Standards Institute broth microdilution

Broth microdilution testing was performed following Clinical and Laboratory Standards Institute (CLSI) protocol with a test inoculum concentration between 0.5 × 10^3^ and 2.5 × 10^3^ colony-forming units (CFU)/mL for *C. auris*, 0.4 × 10^4^–5 × 10^4^ CFU/mL for *A. fumigatus,* and 1 × 10^3^–3 × 10^3^ CFU/mL for *T. indotineae* in RPMI 1640 medium buffered to pH 7.0 with 0.165 M morpholinepropanesulfonic acid buffer. Plates were incubated at 35°C and observed for the presence or absence of growth. The minimum inhibitory concentrations (MICs) of antifungal drugs were determined following CLSI references; antifungal susceptibility testing for *C. auris* was performed according to CLSI document M27, 4th edition (CLSI M27-Ed4), whereas susceptibility testing for *A. fumigatus* and *T. indotineae* was conducted following CLSI document M38, 3rd edition (CLSI M38-Ed3). *Candida parapsilosis* ATCC 22019, *Candida krusei* ATCC 6258, and *A. fumigatus* ATCC 3626 were included as quality control strains. MIC endpoints were determined after incubation for 24 h for *C. auris*, 48 h for *A. fumigatus*, and 96 h for *T. indotineae*.

Resistance was interpreted according to published criteria. For *C. auris*, isolates with fluconazole MICs ≥ 32 µg/mL were considered resistant based on the Centers for Disease Control and Prevention guidance (https://www.cdc.gov/candida-auris/hcp/laboratories/antifungal-susceptibility-testing.html). For *A. fumigatus*, isolates with voriconazole MICs ≥ 2 µg/mL were classified as resistant according to CLSI M61, 2nd edition (25). For *T. indotineae*, resistance was interpreted using the EUCAST epidemiological cutoff values, with isolates exhibiting itraconazole MICs ≥ 0.25 µg/mL, voriconazole MICs ≥ 1 µg/mL, or terbinafine MICs ≥ 1 µg/mL considered resistant (26).

The fungicidal activity of each disinfectant was further evaluated by determining its minimum fungicidal concentration (MFC). MFCs were determined for all 30 fungal isolates using a broth microdilution-based fungicidal assay. Test inocula were prepared in RPMI 1640 medium at final concentrations of 0.5 × 10³–2.5 × 10³ CFU/mL for *C. auris*, 0.4 × 10⁴–5 × 10⁴ CFU/mL for *A. fumigatus*, and 1 × 10³–3 × 10³ CFU/mL for *T. indotineae*. After incubation for 24–96 h, depending on the fungal species, the contents of each well were thoroughly mixed, and 100 μL was plated onto Sabouraud Dextrose Agar plates. Plates were incubated at 30°C for 2–7 days, after which colony growth was assessed. The MFC was defined as the lowest disinfectant concentration that resulted in no visible fungal growth.

### Disinfectant efficacy measurement

Disinfectant efficacy experiments were performed according to the Chinese national standard “Test Method for Bactericidal Effect of Disinfectants in Laboratory” (GB/T 38502-2020). Among the GB/T 38502-2020, the identification experiments for the neutralizing agent are also in accordance with the standards of the European standard EN 13624 for fungicidal activity testing.

Eight disinfectants were evaluated, including 84 Disinfectant solution, dilute glutaral solution, 75% ethanol, iodophor, benzalkonium bromide, Meifute solution, chlorhexidine, and peracetic acid solution. The neutralizers used for each disinfectant were as follows: D/E broth was used for 84 Disinfectant solution, 75% ethanol, iodophor, benzalkonium bromide, and Meifute solution; D/E broth supplemented with 1% sodium bisulfite (NaHSO₃) was used for dilute glutaral solution; a neutralizing solution containing 0.45% NaCl, 3% Tween 80, 0.3% lecithin, and 0.1% cysteine was used for chlorhexidine; and D/E broth supplemented with 0.1% sodium thiosulfate was used for peracetic acid solution.

Neutralizer efficacy was evaluated using four experimental groups. For Group 1 (neutralizer toxicity control), 0.4 mL of standard hard water was added to a test tube containing 4.5 mL of neutralizer. After mixing and incubation at 20 ± 1°C for 5 min, 0.1 mL of fungal suspension (1 × 10⁷ CFU/mL) was added. Following a 10-min contact period, 1 mL of the mixture was transferred onto each of two agar plates for viable fungal enumeration. For Group 2 (disinfectant-neutralizer compatibility control), 0.4 mL of disinfectant was mixed with 4.5 mL of neutralizer and incubated at 20 ± 1°C for 5 min. Subsequently, 0.1 mL of fungal suspension was added, followed by a 10-min contact period. The mixture was then plated (1 mL per plate; two plates per sample) for viable fungal enumeration. For Group 3 (neutralizer validation control), 0.4 mL of standard hard water was mixed with 4.5 mL of dilution solution and incubated at 20 ± 1°C for 5 min. Then, 0.1 mL of fungal suspension was added and allowed to react for 10 min before plating for viable fungal enumeration. For Group 4 (culture medium sterility control), 0.5 mL of dilution solution, 0.5 mL of standard hard water, and 0.5 mL of neutralizer were added to sterile Petri dishes, followed by the addition of 15 mL of the corresponding culture medium. The plates were incubated and monitored for microbial growth.

Disinfectant efficacy experiments were performed according to the Chinese national standard Test Method for Bactericidal Effect of Disinfectants in Laboratory (GB/T 38502-2020). The process of the suspension quantitative kill experiment is basically the same as the European standard EN 13624 for fungicidal activity testing, with three isolates each of *C. auris*, *A. fumigatus*, and *T. indotineae* selected for disinfectant efficacy testing. Susceptible and resistant isolates were included for each species. The disinfectant efficacy assay was performed according to the relevant standard procedures. Briefly, fungal suspensions were prepared at concentrations ranging from 1 × 10⁷ CFU/mL. Disinfectant working solutions were prepared according to the manufacturers’ instructions. For each assay, 0.5 mL of fungal suspension was mixed with 0.5 mL of 3% bovine serum albumin solution as an organic interfering substance to simulate contaminated conditions. The mixture was incubated at 20 ± 1°C for 5 min, followed by the addition of 4 mL of disinfectant solution. The suspension was immediately mixed, and the exposure time was initiated.

After exposure to the disinfectant for 5, 10, or 15 min, 0.5 mL of the reaction mixture was transferred into 4.5 mL of the corresponding neutralizer. After neutralization for 10 min, 1 mL of the neutralized suspension was serially diluted and plated for viable fungal enumeration. In parallel, standard hard water was used instead of disinfectant solution as a positive control under identical conditions. The viable fungal counts were calculated for each treatment group, and the killing efficacy was expressed as the killing logarithm (KL), equivalent to the log₁₀ reduction value, calculated as KL = *N*_0_ *– N_x_*, where *N*_0_ represents the logarithm of the mean viable fungal count in the control group, and *Nₓ* represents the logarithm of the mean viable fungal count after disinfectant treatment. When the product’s specified minimum concentration and the shortest action time are met, as well as 1.5 times the shortest action time, the reduction factor was recorded as >4 log_10_, and the disinfectant was considered qualified. If no fungal colonies grew on the plated surfaces after the disinfectant sterilization test, the reduction factor was recorded as ≥*N*_0_. All experiments were performed in triplicate, and variance statistics were calculated using GraphPad Prism 9.0.

## RESULTS

### Antifungal resistance profiles of 30 strains

Three drug-resistant fungi, *C. auris*, *A. fumigatus*, and *T. indotineae*, were selected to evaluate the efficacy of eight disinfectants commonly used in China. Antifungal susceptibility testing was first profiled across 30 strains before disinfectant evaluation (Table 2).

**TABLE 2:** *In vitro* susceptibilities of 30 strains to itraconazole, voriconazole, posaconazole, fluconazole, and terbinafine as determined by the CLSI M27 and CLSI M38 methods

Species (no. of strains)	Test agent	MIC^*a*^ (range)	MIC_50_^*a*^	MIC_90_^*a*^
*C*. *auris^b^* (10)	Itraconazole	0.06–0.12	0.12	0.12
	Voriconazole	0.015–2	1	2
	Posaconazole	0.015–0.06	0.06	0.06
	Fluconazole	2–>256	256	>256
	Terbinafine	>16	>16	>16
*A. fumigatus^b^* (10)	Itraconazole	0.125–>16	0.25	>16
	Voriconazole	0.25–>8	1	4
	Posaconazole	0.06–1	0.25	1
	Fluconazole	>256	>256	>256
	Terbinafine	0.5–2	2	2
*T*. *indotineae^b^* (10)	Itraconazole	0.125–0.5	0.5	0.5
	Voriconazole	0.06–4	2	2
	Posaconazole	0.06–1	0.5	0.5
	Fluconazole	8–>256	>256	>256
	Terbinafine	0.016–>16	>16	>16
*C*. *krusei* ATCC6258	Itraconazole	0.5		
	Voriconazole	0.5		
	Posaconazole	0.5		
	Fluconazole	16		
	Terbinafine	>16		
*C*. *parapsilosis* ATCC22019	Itraconazole	0.25		
	Voriconazole	0.06		
	Posaconazole	0.125		
	Fluconazole	2		
	Terbinafine	>16		
*A. fumigatus* ATCC3626	Itraconazole	1		
	Voriconazole	0.5		
	Posaconazole	0.125		
	Fluconazole	>64		
	Terbinafine	2		

^*a*^ In micrograms per milliliter.

^*b*^ The minimum inhibitory concentrations (MICs) of antifungal drugs were determined following the CLSI references. Antifungal susceptibility testing for *C. auris* was performed according to the Clinical and Laboratory Standards Institute (CLSI) document M27, 4th edition (CLSI M27-Ed4), whereas susceptibility testing for *A. fumigatus* and *T. indotineae* was conducted following CLSI document M38, 3rd edition (CLSI M38-Ed3). *C. parapsilosis* ATCC 22019, *C. krusei* ATCC 6258, and *A. fumigatus* ATCC 3626 were included as quality control strains. Minimum inhibitory concentration (MIC) endpoints were determined after incubation for 24 h for *C. auris*, 48 h for *A. fumigatus*, and 96 h for *T. indotineae*.

*C. auris* isolates were primarily resistant to fluconazole; *A. fumigatus* isolates were mainly resistant to voriconazole, and *T. indotineae* isolates were predominantly resistant to terbinafine. Ten *C. auris* isolates were not sensitive to terbinafine but were relatively sensitive to itraconazole, voriconazole, and posaconazole. Ten *A. fumigatus* isolates were not sensitive to fluconazole but were relatively sensitive to posaconazole and terbinafine. Some *A. fumigatus* isolates had high MICs for itraconazole and voriconazole. Ten *T. indotineae* isolates were sensitive to itraconazole, voriconazole, and posaconazole. Some *T. indotineae* isolates had high MICs for fluconazole and terbinafine.

### Fungicidal activity of disinfectants at varying concentrations

The disinfectant fungicidal efficacy against all three fungal species was initially assessed using a microbroth dilution method (Table 3). All eight disinfectants demonstrated fungicidal activity against *C. auris*, *A. fumigatus*, and *T. indotineae* when applied at appropriate concentrations, including toward drug-resistant strains.

**TABLE 3:** The minimum fungicidal concentrations of 8 disinfectants against 30 strains *in vitro*

Species (no. of strains)	Test agent	MFC (range)		MFC_50_		MFC_90_	
		Dilution ratios	Concentrations	Dilution ratios	Concentrations	Dilution ratios	Concentrations
*C*. *auris* (10)	84 Disinfectant solution	1:128	0.27–0.36 g/L sodium hypochlorite	1:128	0.27–0.36 g/L sodium hypochlorite	1:128	0.27–0.36 g/L sodium hypochlorite
	Dilute glutaral solution	1:4,096	0.0012 g/L glutaraldehyde, 0.0012 g/L chlorobutyl bromide	1:4,096	0.0012 g/L glutaraldehyde, 0.0012 g/L chlorobutyl bromide	1:4,096	0.0012 g/L glutaraldehyde, 0.0012 g/L chlorobutyl bromide
	75% alcohol	1:16	4.69% ethanol	1:16	4.69% ethanol	1:16	4.69% ethanol
	Iodophor	1:256–1:512	0.008–0.02 g/L of chlorhexidine acetate, 0.0018–0.005 g/L of iodine, 0.12%–0.27% ethanol	1:512	0.008–0.01g/L of chlorhexidine acetate, 0.0018–0.0023 g/L of iodine, 0.12%–0.14% ethanol	1:256	0.016–0.02 g/L of chlorhexidine acetate, 0.004–0.005 g/L of iodine, 0.23%–0.27% ethanol
	Benzalkonium bromide	1:2,048–1:4,096	0.0073–0.015 g/L benzalkonium bromide	1:4,096	0.0073 g/L benzalkonium bromide	1:2,048	0.015 g/L benzalkonium bromide
	Meifute	1:8,192	0.00,027 g/L benzalkonium chloride, silver ions, alkyl glucoside, menthol, citric acid, lactic acid, glycerin, coconut fatty acid amine	1:8,192	0.00,027 g/L benzalkonium chloride, silver ions, alkyl glucoside, menthol, citric acid, lactic acid, glycerin, coconut fatty acid amine	1:8,192	0.00,027 g/L benzalkonium chloride, silver ions, alkyl glucoside, menthol, citric acid, lactic acid, glycerin, coconut fatty acid amine
	Chlorhexidine	1:16–1:32	0.0063%–0.0125% chlorhexidine gluconate	1:32	0.0063% chlorhexidine gluconate	1:16	0.0125% chlorhexidine gluconate
	Peracetic acid	1:512–1:1,024	0.015%–0.035% peracetic acid, 0.012%–0.028% acetic acid, 0.024%–0.055% hydrogen peroxide	1:1,024	0.015%–0.018% peracetic acid, 0.012%–0014% acetic acid, 0.024%–0.028% hydrogen peroxide	1:512	0.029%–0.035% peracetic acid, 0.023%–0.028% acetic acid, 0.047%–0.055% hydrogen peroxide
*A*. *fumigatus* (10)	84 Disinfectant solution	1:32–1:64	0.53–1.44 g/L sodium hypochlorite	1:32	0.53–1.44 g/L sodium hypochlorite	1:32	0.53–1.44 g/L sodium hypochlorite
	Dilute glutaral solution	1:4,096–1:8,192	0.0006 g/L glutaraldehyde, 0.0006 g/L chlorobutyl bromide-0.0012 g/L glutaraldehyde, 0.0012 g/L chlorobutyl bromide	1:8,192	0.0006 g/L glutaraldehyde, 0.0006 g/L chlorobutyl bromide	1:4,096	0.0012 g/L glutaraldehyde, 0.0012 g/L chlorobutyl bromide
	75% alcohol	1:8–1:32	2.34% ethanol-9.38% ethanol	1:32	2.34% ethanol	1:16	4.69% ethanol
	Iodophor	1:128–1:256	4.2–5.2 g/L of chlorhexidine acetate, 0.9–1.2 g/L of iodine, 60%–70% ethanol	1:128	0.03–0.04 g/L of chlorhexidine acetate, 0.007–0.009 g/L of iodine, 0.47%–0.55% ethanol	1:128	0.03–0.04 g/L of chlorhexidine acetate, 0.007–0.009 g/L of iodine, 0.47%–0.55% ethanol
	Benzalkonium bromide	1:2,048–1:4,096	0.0073–0.015 g/L benzalkonium bromide	1:4,096	0.0073 g/L benzalkonium bromide	1:2,048	0.015g/L benzalkonium bromide
	Meifute	1:1,024	0.0022 g/L benzalkonium chloride, silver ions, alkyl glucoside, menthol, citric acid, lactic acid, glycerin, coconut fatty acid amine	1:1,024	0.0022 g/L benzalkonium chloride, silver ions, alkyl glucoside, menthol, citric acid, lactic acid, glycerin, coconut fatty acid amine	1:1,024	0.0022 g/L benzalkonium chloride, silver ions, alkyl glucoside, menthol, citric acid, lactic acid, glycerin, coconut fatty acid amine
	Chlorhexidine	1:16–1:32	0.0063%–0.0125% chlorhexidine gluconate	1:32	0.0063% chlorhexidine gluconate	1:16	0.0125% chlorhexidine gluconate
	Peracetic acid	1:1,024	0.015%–0.018% peracetic acid, 0.012%–0014% acetic acid, 0.024%–0.028% hydrogen peroxide	1:1,024	0.015%–0.018% peracetic acid, 0.012%0014% acetic acid, 0.024%–0.028% hydrogen peroxide	1:1,024	0.015%–0.018% peracetic acid, 0.012%–0014% acetic acid, 0.024%–0.028% hydrogen peroxide
*T*. *indotineae* (10)	84 Disinfectant solution	1:128	0.27–0.36 g/L sodium hypochlorite	1:128	0.27–0.36 g/L sodium hypochlorite	1:128	0.27–0.36 g/L sodium hypochlorite
	Dilute glutaral solution	1:8,192–1:16,384	0.0003 g/L glutaraldehyde, 0.0003 g/L chlorobutyl bromide-0.0006 g/L glutaraldehyde, 0.0006 g/L chlorobutyl bromide	1:16,384	0.0003 g/L glutaraldehyde, 0.0003 g/L chlorobutyl bromide	1:8,192	0.0006 g/L glutaraldehyde, 0.0006 g/L chlorobutyl bromide
	75% alcohol	1:2–1:4	18.75% ethanol- 37.5% ethanol	1:4	18.75% ethanol	1:4	18.75% ethanol
	Iodophor	1:512	0.008–0.01 g/L of chlorhexidine acetate, 0.0018–0.0023 g/L of iodine, 0.12%–0.14% ethanol	1:512	0.008–0.01 g/L of chlorhexidine acetate, 0.0018–0.0023 g/L of iodine, 0.12%–0.14% ethanol	1:512	0.008–0.01 g/L of chlorhexidine acetate, 0.0018–0.0023 g/L of iodine, 0.12%–0.14% ethanol
	Benzalkonium bromide	1:2,048	0.015 g/L benzalkonium bromide	1:2,048	0.015 g/L benzalkonium bromide	1:2,048	0.015 g/L benzalkonium bromide
	Meifute	1:2,048	0.0011 g/L benzalkonium chloride, silver ions, alkyl glucoside, menthol, citric acid, lactic acid, glycerin, coconut fatty acid amine	1:2,048	0.0011 g/L benzalkonium chloride, silver ions, alkyl glucoside, menthol, citric acid, lactic acid, glycerin, coconut fatty acid amine	1:2,048	0.0011 g/L benzalkonium chloride, silver ions, alkyl glucoside, menthol, citric acid, lactic acid, glycerin, coconut fatty acid amine
	Chlorhexidine	1:64	0.003% chlorhexidine gluconate	1:64	0.003% chlorhexidine gluconate	1:64	0.003% chlorhexidine gluconate
	Peracetic acid	1:8,192	0.0018%–0.0022% peracetic acid, 0.0015%–0.0018% acetic acid, 0.0030%–0.0034% hydrogen peroxide	1:8,192	0.0018%–0.0022% peracetic acid, 0.0015%–0.0018% acetic acid, 0.0030%0.0034% hydrogen peroxide	1:8,192	0.0018%–0.0022% peracetic acid, 0.0015%–0.0018% acetic acid, 0.0030%–0.0034% hydrogen peroxide

### Kill rate of disinfectants at recommended working concentrations

The neutralizer validation experiments confirmed that the selected neutralizers effectively eliminated the residual activity of the corresponding disinfectants. After neutralization, viable fungal counts recovered from treated samples showed no significant differences compared with the control groups without disinfectant exposure.

To evaluate the practical efficacy of disinfectants, drug-resistant and -sensitive fungal strains for all three species were tested at the manufacturer-specified working concentrations (Table 4, Table S1). The proportion of drug-resistant strains in 30 isolates from *C. auris* and *T. indotineae* in China is higher, while that of *A. fumigatus* drug-resistant strains is relatively low (within 10%). In the disinfectant killing test, the following strains were used: *A. fumigatus* (one resistant and two susceptible), *T. indotineae* (two resistant and one susceptible), and *C. auris* (two resistant and one susceptible). We examined the mutations in the SQLE region of 10 *T. indotineae* strains and found that 1 strain had an Ala448Thr mutation, while the remaining nine had a Phe397Leu mutation.

**TABLE 4:** The disinfectant’s efficacy in killing fungi

Species	Time	Reduction factor^*a*^ (The log_10_ reduction value)							
		I^*b*^	II^*b*^	III^*b*^	IV^*b*^	V^*b*^	VI^*b*^	VII^*b*^	VIII^*b*^
*C. auris* 52467 (Resistant strain)	5 min	0.001 ± 0.02	0.019 ± 0.023	≥5 ± 0	≥5 ± 0	≥5 ± 0	≥5 ± 0	≥5 ± 0	≥5 ± 0
	10 min	0.0012 ± 0.013	0.011 ± 0.063	≥5 ± 0	≥5 ± 0	≥5 ± 0	≥5 ± 0	≥5 ± 0	≥5 ± 0
	15 min	0.0098 ± 0.017	0.014 ± 0.025	≥5 ± 0	≥5 ± 0	≥5 ± 0	≥5 ± 0	≥5 ± 0	≥5 ± 0
*C. auris* 52321 (Resistant strain)	5 min	0.0054 ± 0.021	0.0018 ± 0.041	≥5 ± 0	≥5 ± 0	≥5 ± 0	≥5 ± 0	≥5 ± 0	≥5 ± 0
	10 min	0.0028 ± 0.014	0.01 ± 0.049	≥5 ± 0	≥5 ± 0	≥5 ± 0	≥5 ± 0	≥5 ± 0	≥5 ± 0
	15 min	0.021 ± 0.033	0.026 ± 0.034	≥5 ± 0	≥5 ± 0	≥5 ± 0	≥5 ± 0	≥5 ± 0	≥5 ± 0
*C. auris* 52469 (Sensitive strain)	5 min	0.0057 ± 0.023	0.0027 ± 0.036	≥5 ± 0	≥5 ± 0	≥5 ± 0	≥5 ± 0	≥5 ± 0	≥5 ± 0
	10 min	0.0092 ± 0.03	0.0073 ± 0.021	≥5 ± 0	≥5 ± 0	≥5 ± 0	≥5 ± 0	≥5 ± 0	≥5 ± 0
	15 min	0.011 ± 0.015	0.013 ± 0.017	≥5 ± 0	≥5 ± 0	≥5 ± 0	≥5 ± 0	≥5 ± 0	≥5 ± 0
*A. fumigatus* 04270 (Resistant strain)	5 min	0.015 ± 0.017	0.022 ± 0.036	≥5 ± 0	≥5 ± 0	≥5 ± 0	≥5 ± 0	≥5 ± 0	≥5 ± 0
	10 min	0.018 ± 0.014	0.24 ± 0.06	≥5 ± 0	≥5 ± 0	≥5 ± 0	≥5 ± 0	≥5 ± 0	≥5 ± 0
	15 min	0.00034 ± 0.037	0.95 ± 0.083	≥5 ± 0	≥5 ± 0	≥5 ± 0	≥5 ± 0	≥5 ± 0	≥5 ± 0
*A. A. fumigatus* 04860 (Sensitive strain)	5 min	0.0095 ± 0.031	0.0085 ± 0.026	≥5 ± 0	≥5 ± 0	≥5 ± 0	≥5 ± 0	≥5 ± 0	≥5 ± 0
	10 min	0.0034 ± 0.042	0.23 ± 0.05	≥5 ± 0	≥5 ± 0	≥5 ± 0	≥5 ± 0	≥5 ± 0	≥5 ± 0
	15 min	0.013 ± 0.03	0.90 ± 0.12	≥5 ± 0	≥5 ± 0	≥5 ± 0	≥5 ± 0	≥5 ± 0	≥5 ± 0
*A. fumigatus* 04811 (Sensitive strain)	5 min	0.0021 ± 0.022	0.0037 ± 0.016	≥5 ± 0	≥5 ± 0	≥5 ± 0	≥5 ± 0	≥5 ± 0	≥5 ± 0
	10 min	0.0016 ± 0.013	0.24 ± 0.026	≥5 ± 0	≥5 ± 0	≥5 ± 0	≥5 ± 0	≥5 ± 0	≥5 ± 0
	15 min	0.0015 ± 0.014	0.91 ± 0.14	≥5 ± 0	≥5 ± 0	≥5 ± 0	≥5 ± 0	≥5 ± 0	≥5 ± 0
*T. indotineae* 05770 (Resistant strain, Phe397Leu)	5 min	0.013 ± 0.041	0.0068 ± 0.028	≥5 ± 0	≥5 ± 0	0.26 ± 0.056	≥5 ± 0	≥5 ± 0	≥5 ± 0
	10 min	0.00074 ± 0.022	0.0011 ± 0.033	≥5 ± 0	≥5 ± 0	0.73 ± 0.09	≥5 ± 0	≥5 ± 0	≥5 ± 0
	15 min	0.005 ± 0.038	0.0024 ± 0.055	≥5 ± 0	≥5 ± 0	1.009 ± 0.056	≥5 ± 0	≥5 ± 0	≥5 ± 0
*T. indotineae* 03895 (Sensitive strain Ala448Thr)	5 min	0.0013 ± 0.051	0 ± 0.026	≥5 ± 0	≥5 ± 0	0.22 ± 0.069	≥5 ± 0	≥5 ± 0	≥5 ± 0
	10 min	0.012 ± 0.048	0.0077 ± 0.037	≥5 ± 0	≥5 ± 0	0.78 ± 0.094	≥5 ± 0	≥5 ± 0	≥5 ± 0
	15 min	0.013 ± 0.045	0.016 ± 0.44	≥5 ± 0	≥5 ± 0	1.05 ± 0.093	≥5 ± 0	≥5 ± 0	≥5 ± 0
*T. indotineae* 04782 (Resistant strain, Phe397Leu)	5 min	0.0029 ± 0.04	0.007 ± 0.0065	≥5 ± 0	≥5 ± 0	0.24 ± 0.021	≥5 ± 0	≥5 ± 0	≥5 ± 0
	10 min	0.01 ± 0.007	0.01 ± 0.016	≥5 ± 0	≥5 ± 0	0.60 ± 0.071	≥5 ± 0	≥5 ± 0	≥5 ± 0
	15 min	0.0066 ± 0.025	0.012 ± 0.015	≥5 ± 0	≥5 ± 0	0.95 ± 0.092	≥5 ± 0	≥5 ± 0	≥5 ± 0

^*a*^ The values in the table represent KL ± SD. Formula: KL = *N*_0_−*N*_*x*_. KL represents the Reduction factor, *N*_0_ is the logarithmic form of the average viable fungi count of the control group, and *N*_*x*_ is the logarithmic form of the viable fungi count of the experimental group.

^*b*^ The names of the eight disinfectants are as follows: 84 Disinfectant solution (I), Dilute glutaral solution (II), 75% Ethanol (III), Iodophor (IV), Benzalkonium bromide (V), Meifute solution (VI), Chlorhexidine (VII), and Peracetic Acid Solution (VIII).

Fungal killing rates over 5, 10, and 15 min of exposure are summarized below. For three *C. auris* strains, 84 Disinfectant and dilute glutaral solutions exhibited limited fungicidal activity, with no substantial reduction in viable fungal counts observed at any of the tested exposure times. In contrast, the remaining six disinfectants achieved complete fungal inactivation, resulting in a ≥5 log₁₀ reduction in viable counts within 5 min. Similar results were observed for the 3 *A. fumigatus* strains; 84 Disinfectant and dilute glutaral solutions showed minimal fungicidal activity, whereas the other 6 disinfectants achieved complete fungal inactivation (≥5 log₁₀ reduction) within 5 min. For the 3 *T. indotineae* strains, 84 Disinfectant solution, dilute glutaral solution, and benzalkonium bromide demonstrated limited fungicidal activity. The remaining five disinfectants achieved complete fungal inactivation, with a ≥ 5 log₁₀ reduction in viable counts within 5 min of exposure.

Overall, five disinfectants, when applied at their working concentrations, were able to eliminate all three fungi within 5 min of exposure, whether they were drug-resistant or susceptible, highlighting their high efficacy in practical disinfection scenarios.

## DISCUSSION

This study aimed to evaluate the germicidal efficacy of disinfectants against three types of fungi that pose significant treatment challenges owing to emerging drug resistance. A few years ago, China only focused on drug-resistant *C. auris* and *A. fumigatus* (27–29); it was not until the first reported local infection of *T. indotineae* in 2022 (30) that attention shifted to the drug resistance of superficial fungi. These findings underscore the potential for both deep and superficial drug-resistant fungi to cause nosocomial infections or be involved in household transmission (31). It is critical to implement thorough disinfection of drug-resistant fungi in both domestic and medical environments by selecting the most appropriate disinfectants for these settings.

Testing in this study followed the GB/T 38502-2020 standard; the operational requirements for neutralizing agent identification and suspension quantitative killing testing were similar to those of the EN 13624 standard. The sterilization threshold EN 1275 is ≥4 log₁₀, which is the same as the GB/T 38502-2020 standard used here. This study only examined fungal suspensions (planktonic cells), so no carrier-surface claim is supported.

In China, chlorine-based disinfectants are commonly used in hospitals and drinking water systems (32–34). However, reported kill rates of chlorine disinfectants against fungi such as *Aspergillus* and *Penicillium* often do not reach 100% (35). The present study produced similar findings: the chlorine-based 84 Disinfectant solution did not eradicate the 3 tested fungi at manufacturer-specified working concentrations. Similarly, the aldehyde-based disinfectant dilute glutaral solution was also unable to eradicate three fungi when used at the label-specified working concentration for 15 min, and benzalkonium bromide, when used at the label-specified working concentration for 15 min, was unable to eradicate *T. indotineae*. These findings suggest that these three disinfectants should not be relied upon at recommended working concentrations in environments where drug-resistant fungi are present.

Iodophor solution is a mixture of three active formulations (chlorhexidine acetate, iodine, and ethanol); although effective, it has a distinct brownish yellow color that may stain, making it more suitable for disinfecting patient skin rather than environmental surfaces (36). In contrast, 75% ethanol achieved complete inactivation of all three drug-resistant fungi within 5 min and is recommended for use in areas free from open flames or flammable substances (37). In another study conducted in 2026, the ethanol-based disinfectant (40%) effectively achieved a 4 log_10_ reduction for all tested *Candida* strains. When the ethanol concentration is as low as 30%, it causes different degrees of reduction in different *C. auris* strains. Additionally, the use of different concentrations of quaternary ammonium compound (QAC)-based disinfectant showed more substantial inter-species and inter-clade differences in *C. auris*; it reached a 4 log_10_ reduction with lower concentrations in *C. auris* strains of clades I (0.125% QAC), III (0.125% QAC), and IV (0.1% QAC) (20).

This study did not focus on the evolutionary branches of the strains, instead focusing only on drug sensitivity. During disinfectant testing, both resistant and sensitive strains of each species were examined. The three strains of *C. auris* in this study were all tested at the commercial working concentrations indicated for each of the eight disinfectants, with consistent results across strains. Benzalkonium bromide and Meifute solution are also QAC-based disinfectants. The working concentrations used for each were 0.2% and 0.225%, respectively, achieving a reduction of ≥5 log_10_ for the three fungal strains.

Meifute and peracetic acid solutions were able to achieve a ≥5 log₁₀ reduction against all three drug-resistant fungi within 5 min. Even when diluted ≥1,000-fold, they maintained strong fungicidal activity. Based on MFC advantages, suspension data, and applicable scenarios, these two compounds demonstrate strong potential as effective disinfectants against the three drug-resistant fungi tested. Their high fungicidal efficacy may contribute to reducing environmental contamination and limiting the transmission of drug-resistant fungal pathogens. However, *C. auris* and *A. fumigatus* are known to form biofilms on various surfaces, which may reduce the effectiveness of conventional disinfection procedures. Future studies are needed to further evaluate the activity of these disinfectants against fungal biofilms and determine their effectiveness under more complex environmental conditions. At present, the suspension data alone are insufficient to support usage recommendations for specific environments; further carrier testing and in-use field validation will be necessary. Therefore, the current study provides an initial experimental basis and data for the future development of effective disinfectants that will be suitable for use in clinical or other environments against drug-resistant fungi.

## Data Availability

All sequences of 30 isolates were submitted to GenBank under accession numbers PZ007869 to PZ007898 for internal transcribed spacer sequences and PZ007900 to PZ007929 for the large subunit. The squalene epoxidase (SQLE) gene sequences of 10 T. indotineae were submitted to GenBank under accession numbers PZ961926–PZ961935.

## SUPPLEMENTAL MATERIAL

Table S1 spectrum.01055-26-s0001.xlsx Raw colony counts and measured control counts for each isolate and replicate.

ASM does not own the copyrights to Supplemental Material that may be linked to, or accessed through, an article. The authors have granted ASM a non-exclusive, world-wide license to publish the Supplemental Material files. Please contact the corresponding author directly for reuse.

## References

[1] Żelechowska P, Pastwińska J, Brzezińska-Błaszczyk E, Agier J. 2021. Do mast cells contribute to the antifungal host defense? Cells 10:2510. doi:10.3390/cells10102510 PMC8534142 34685489

[2] Buzzini P, Turchetti B, Yurkov A. 2018. Extremophilic yeasts: the toughest yeasts around? Yeast 35:487–497. doi:10.1002/yea.3314 29577430

[3] Hibbett D, Nagy LG, Nilsson RH. 2025. Fungal diversity, evolution, and classification. Curr Biol 35:R463–R469. doi:10.1016/j.cub.2025.01.053 40494297

[4] Strickland AB, Shi M. 2021. Mechanisms of fungal dissemination. Cell Mol Life Sci 78:3219–3238. doi:10.1007/s00018-020-03736-z PMC8044058 33449153

[5] Groll AH, Walsh TJ. 2001. Uncommon opportunistic fungi: new nosocomial threats. Clin Microbiol Infect 7 Suppl 2:8–24. doi:10.1111/j.1469-0691.2001.tb00005.x 11525222

[6] Harris AD. 2008. How important is the environment in the emergence of nosocomial antimicrobial-resistant bacteria? Clin Infect Dis 46:686–688. doi:10.1086/527395 18230046

[7] Luo K, Tang J, Qu Y, Yang X, Zhang L, Chen Z, Kuang L, Su M, Mu D. 2021. Nosocomial infection by Klebsiella pneumoniae among neonates: a molecular epidemiological study. J Hosp Infect 108:174–180. doi:10.1016/j.jhin.2020.11.028 33290814

[8] Facciolà A, Pellicanò GF, Visalli G, Paolucci IA, Venanzi Rullo E, Ceccarelli M, D’Aleo F, Di Pietro A, Squeri R, Nunnari G, La Fauci V. 2019. The role of the hospital environment in the healthcare-associated infections: a general review of the literature. Eur Rev Med Pharmacol Sci 23:1266–1278. doi:10.26355/eurrev_201902_17020 30779096

[9] Rhodes J, Fisher MC. 2019. Global epidemiology of emerging *Candida auris*. Curr Opin Microbiol 52:84–89. doi:10.1016/j.mib.2019.05.008 31279224

[10] Roberts SC, Zembower TR, Ozer EA, Qi C. 2021. Genetic evaluation of nosocomial *Candida auris* transmission. J Clin Microbiol 59:e02252-20. doi:10.1128/JCM.02252-20 PMC8092739 33472901

[11] Diba K, Jangi F, Makhdoomi K, Moshiri N, Mansouri F. 2019. *Aspergillus* diversity in the environments of nosocomial infection cases at a university hospital. J Med Life 12:128–132. doi:10.25122/jml-2018-0057 PMC6685303 31406513

[12] Dahiya S, Chhillar AK, Sharma N, Choudhary P, Punia A, Balhara M, Kaushik K, Parmar VS. 2020. *Candida auris* and nosocomial infection. Curr Drug Targets 21:365–373. doi:10.2174/1389450120666190924155631 31549952

[13] Perdelli F, Cristina ML, Sartini M, Spagnolo AM, Dallera M, Ottria G, Lombardi R, Grimaldi M, Orlando P. 2006. Fungal contamination in hospital environments. Infect Control Hosp Epidemiol 27:44–47. doi:10.1086/499149 16418986

[14] Fabrizio G, Sivori F, Cavallo I, Truglio M, Toma L, Sperati F, Francalancia M, Obregon F, Pamparau L, Kovacs D, Pimpinelli F, Di Domenico EG. 2024. Efficacy of sodium hypochlorite in overcoming antimicrobial resistance and eradicating biofilms in clinical pathogens from pressure ulcers. Front Microbiol 15:1432883. doi:10.3389/fmicb.2024.1432883 PMC11266179 39050624

[15] Tyagi SP, Sinha DJ, Garg P, Singh UP, Mishra CC, Nagpal R. 2013. Comparison of antimicrobial efficacy of propolis, *Morinda citrifolia*, *Azadirachta indica* (Neem) and 5% sodium hypochlorite on *Candida albicans* biofilm formed on tooth substrate: an *in-vitro* study. J Conserv Dent 16:532–535. doi:10.4103/0972-0707.120973 PMC3842722 24347888

[16] Lopes LG, Csonka LA, Castellane JAS, Oliveira AW, de Almeida-Júnior S, Furtado RA, Tararam C, Levy LO, Crivellenti LZ, Moretti ML, Giannini MJSM, Pires RH. 2021. Disinfectants in a hemodialysis setting: antifungal activity against *Aspergillus* and fusarium planktonic and biofilm cells and the effect of commercial peracetic acid residual in mice. Front Cell Infect Microbiol 11:663741. doi:10.3389/fcimb.2021.663741 PMC8116949 33996634

[17] Moriello KA. 2015. Kennel disinfectants for *Microsporum canis* and *Trichophyton* sp. Vet Med Int 2015:853937. doi:10.1155/2015/853937 PMC4339702 25763290

[18] Gupta AK, Ahmad I, Summerbell RC. 2002. Fungicidal activities of commonly used disinfectants and antifungal pharmaceutical spray preparations against clinical strains of *Aspergillus* and *Candida* species. Med Mycol 40:201–208. doi:10.1080/mmy.40.2.201.208 12058733

[19] Gasparto AK, Baltazar LM, Gouveia LF, da Silva CM, Byrro RMD, Rachid MA, Cunha Júnior A da S, de Resende-Stoianoff MA, de Fátima A, Santos DA. 2015. 2-(benzylideneamino)phenol: a promising hydroxyaldimine with potent activity against dermatophytoses. Mycopathologia 179:243–251. doi:10.1007/s11046-014-9850-5 25515245

[20] Lang A, Gabriel H, Meister TL, Meis JF, Steinmann E, Brill FHH, Steinmann J. 2026. Differential susceptibility of *Candida* (Candidozyma) auris clades to surface disinfectants. J Hosp Infect 167:91–99. doi:10.1016/j.jhin.2025.10.003 41101386

[21] Stauf R, Todt D, Steinmann E, Rath PM, Gabriel H, Steinmann J, Brill FHH. 2019. *In-vitro* activity of active ingredients of disinfectants against drug-resistant fungi. J Hosp Infect 103:468–473. doi:10.1016/j.jhin.2019.07.013 31356854

[22] Rabby MRI, Hossain MS, Chowdhury NN, Akter F, Razu MH, Ahmed ZB, Khan M, El-Gendy N. 2025. Comparative evaluation of a disinfectant formulation comprising hydrogen peroxide, peracetic ACID, and acetic ACID against *Aspergillus niger*, *Escherichia coli*, and *Staphylococcus aureus* on various surfaces in comparison to other disinfectants. Int J Microbiol 2025:2602317. doi:10.1155/ijm/2602317 PMC12213054 40599609

[23] WHO. 2022. WHO fungal priority pathogens list to guide research, development and public health action. https://www.who.int/publications/i/item/9789240060241.

[24] Kong X, Tang C, Singh A, Ahmed SA, Al-Hatmi AMS, Chowdhary A, Nenoff P, Gräser Y, Hainsworth S, Zhan P, Meis JF, Verweij PE, Liu W, de Hoog GS. 2021. Antifungal susceptibility and mutations in the squalene epoxidase gene in dermatophytes of the trichophyton mentagrophytes species complex. Antimicrob Agents Chemother 65:e00056-21. doi:10.1128/AAC.00056-21 PMC8284460 33972254

[25] CLSI. 2020. Performance standards for antifungal susceptibility testing of filamentous fungi, p 1–28

[26] EUCAST. 2025. Overview of antifungal ECOFFs and clinical breakpoints for yeasts, moulds and dermatophytes using the EUCAST E.Def 7.4, E.Def 9.4 and E.Def 11.0 procedures

[27] Liu M, Zeng R, Zhang L, Li D, Lv G, Shen Y, Zheng H, Zhang Q, Zhao J, Zheng N, Liu W. 2015. Multiple cyp51A-based mechanisms identified in azole-resistant isolates of *Aspergillus fumigatus* from China. Antimicrob Agents Chemother 59:4321–4325. doi:10.1128/AAC.00003-15 PMC4468671 25896700

[28] Du H, Bing J, Nobile CJ, Huang G. 2022. Candida auris infections in China. Virulence 13:589–591. doi: 10.1080/21505594.2022.2054120 10.1080/21505594.2022.2054120 PMC9037403 35441578

[29] Cao D, Wu R, Dong S, Wang F, Ju C, Yu S, Xu S, Fang H, Yu Y. 2020. Five-year survey (2014 to 2018) of azole resistance in environmental *Aspergillus fumigatus* isolates from China. Antimicrob Agents Chemother 64:e00904–20. doi:10.1128/AAC.00904-20 PMC7508624 32718960

[30] Kong X, Song G, Mei H, Zheng H, Tang C, de Hoog S, Li X, She X, Liu W, Liang G. 2023. The domestic isolation of terbinafine- and itraconazole-resistant trichophyton indotineae In Chinese mainland. Mycopathologia 188:383–393. doi:10.1007/s11046-023-00761-x 37335400

[31] Xie W, Liang G, Kong X, Tsui CKM, She X, Liu W, Li X. 2025. Genomic epidemiology and antifungal resistance of emerging *Trichophyton indotineae* in China. Emerg Microbes Infect 14:2498571. doi:10.1080/22221751.2025.2498571 PMC12077451 40293476

[32] Xu M-Y, Lin Y-L, Zhang T-Y, Hu C-Y, Tang Y-L, Deng J, Xu B. 2022. Chlorine dioxide-based oxidation processes for water purification：a review. J Hazard Mater 436:129195. doi:10.1016/j.jhazmat.2022.129195 35739725

[33] Zhang X, Wei Y, Zhao G, He M, Sun J, Zeng W. 2023. Coronavirus disease 2019: repeated immersion of chlorine-containing disinfectants has adverse effects on goggles. Front Public Health 11:1016938. doi:10.3389/fpubh.2023.1016938 PMC9895403 36741956

[34] Wang J, Shen J, Ye D, Yan X, Zhang Y, Yang W, Li X, Wang J, Zhang L, Pan L. 2020. Disinfection technology of hospital wastes and wastewater: suggestions for disinfection strategy during coronavirus Disease 2019 (COVID-19) pandemic in China. Environmental Pollution 262:114665. doi:10.1016/j.envpol.2020.114665 PMC7194566 32443202

[35] Xu X, Cao R, Li K, Wan Q, Wu G, Lin Y, Huang T, Wen G. 2022. The protective role and mechanism of melanin for *Aspergillus niger* and *Aspergillus flavus* against chlorine-based disinfectants. Water Res 223:119039. doi:10.1016/j.watres.2022.119039 36084430

[36] Gussin GM, McKinnell JA, Singh RD, Miller LG, Kleinman K, Saavedra R, Tjoa T, Gohil SK, Catuna TD, Heim LT, et al.. 2024. Reducing hospitalizations and multidrug-resistant organisms via regional decolonization in hospitals and nursing homes. JAMA 331:1544–1557. doi:10.1001/jama.2024.2759 PMC10985619 38557703

[37] Spigelman AD, Swan JR. 2005. Skin antiseptics and the risk of operating theatre fires. ANZ J Surg 75:556–558. doi:10.1111/j.1445-2197.2005.03429.x 15972046

